# Orientation Invariant Sensorimotor Object Recognition Using Cortical Grid Cells

**DOI:** 10.3389/fncir.2021.738137

**Published:** 2022-01-26

**Authors:** Kalvyn Roux, David van den Heever

**Affiliations:** ^1^BERG, Department of Mechanical and Mechatronic Engineering, Stellenbosch University, Stellenbosch, South Africa; ^2^Department of Agricultural and Biological Engineering, Mississippi State University, Starkville, MS, United States

**Keywords:** neocortex, sensorimotor learning, grid cells, hierarchical temporal memory, object recognition, orientation invariance

## Abstract

Grid cells enable efficient modeling of locations and movement through path integration. Recent work suggests that the brain might use similar mechanisms to learn the structure of objects and environments through sensorimotor processing. This work is extended in our network to support sensor orientations relative to learned allocentric object representations. The proposed mechanism enables object representations to be learned through sensorimotor sequences, and inference of these learned object representations from novel sensorimotor sequences produced by rotated objects through path integration. The model proposes that orientation-selective cells are present in each column in the neocortex, and provides a biologically plausible implementation that echoes experimental measurements and fits in with theoretical predictions of previous studies.

## 1. Introduction

We perceive the world around us through sensory experience, interpreting bottom-up sensory input with internal top-down expectations (Gilbert and Sigman, 2007). Sensory input continuously changes according to the environment changing and/or the respective sensors moving relative to the environment. Saccades are an example of stable perceptions despite the continuous movement of our sensors (eyes) during sensory experience. This stable perception is invariant to the order in which the sequence of samples of the environment occurs, and is refined through movement of sensors relative to objects (as we look around). These stable representations result from correct predictions of upcoming sensory input, by including upcoming self-generated movements in conjunction with the stream of sensory inputs when calculating expectations (Killian et al., 2012, 2015). This enables the brain to filter out meaningless sensory inputs. Neural representations therefore need to take into account both object-centric (also known as *allocentric*) and body-centric (also known as *egocentric*) locations of sensors in order to accommodate the necessary invariances needed for sensory inputs (Burgess, 2006).

In the brain, neural responsiveness to sensory input and spatial location signals can provide insight into how neural activity represents meaning. Grid cells might give a neurally plausible solution to the integration of *sensorimotor* information over time, by providing a location signal for sensory inputs. Cells residing in the hippocampal complex and entorhinal cortex (deeply embedded in the temporal lobe) have been studied in depth in recent years, and are involved in learning and memory and the aforementioned location signal (Moser et al., 2008; Kropff et al., 2015; Wang et al., 2020). Some of the most well-known cells involved in neural spatial representations are place cells (O'Keefe and Dostrovsky, 1971), grid cells (Hafting et al., 2005), head direction cells (Taube et al., 1990), and border cells (Lever et al., 2009) among others, in addition to many types of conjunctive cells that show combined properties of these spatially selective cells (Hardcastle et al., 2017). Their activity over time is updated via a combination of self-generated movement efference copies and sensory based landmarks (Campbell et al., 2018). The predictive nature of the system is also indicated by the selective activity of neuron populations when known locations provide different sensory inputs than before (Tsao et al., 2013).

Some have motivated that grid cell-like computations could be used to construct allocentric object representations in various sensory modalities (Hawkins et al., 2019). Previous work (Lewis et al., 2019) demonstrated how sensorimotor object recognition could be implemented in the neocortex using such computations, and subsequently proposed equivalent mechanisms to grid cells in the neocortex.

The integration of sensorimotor processing over time (also known as *path integration*), to update neural representations is a useful property to have. It provides the benefit of path independent localization for the animal i.e., the same grid cells in a module will be active for a location even if different paths have been taken to get there. Errors can occur during path integration, so learned sensory landmarks of individual environments are used as anchors to mitigate them in the spatial system defined by grid cells (McNaughton et al., 2006). Path integration can also be seen as a form of generalization with respect to spatial representations for unseen locations. The anchoring of grid cells to specific environments also suggest that representations unique to that environment are able to be formed despite possibly meaning something different in another environment (Fiete et al., 2008; Sreenivasan and Fiete, 2011).

The combination of these properties of grid cells helps to broadly define how grid cells can represent an environment, as well as the relationship to other environments. An environment can be seen as a unique location subspace within the previously mentioned representational capacity of combined grid cell modules. As seen from these discrete grid cell representations, an environment can be defined simply as a set of location representations related to each other by path integration (or by being connected via movement vectors). These locations include all possible locations in that environment (including the ones that have not been visited), and landmarks for that specific environment are also associated with these possible locations. Previously mentioned path integration properties also hold for new environments, since each grid cell module independently integrates motion vectors. Readers are directed to Lewis et al. (2019) for a more in depth discussion of the grid cell computations explored here and used in the proposed model.

In the context of the main contribution of this paper, orientation invariance, grid cells have also been shown to be involved in virtual navigation in a similar fashion to their normal function in the entorhinal cortex (Horner et al., 2016). Mental navigation and planning could therefore involve the same mechanisms used for spatial representation in the entorhinal cortex for virtual navigation signals such as movement with grid cells, or orientation with head direction cells (Byrne et al., 2007).

Prior work showed how a network of cortical columns and different neocortical layers can process a sensorimotor sequence to learn allocentric object representations composed of synthetic features. Specialized neurons in each layer of the network can learn to associate locations to features by considering sensor movement and sensations together. These associations could then be used to generate predictions of sensory inputs, by using the integration of movement to object recognition to generalize over novel sensorimotor sequences (Hawkins et al., 2017). However, the mechanism for location signals in this model was lacking in biological plausibility. This was addressed and was dealt with by adding an additional grid cell-based location layer in Lewis et al. (2019). The neural activity used throughout these models is represented by sparse distributed representations, which are high dimensional binary vectors with a only small number of active nodes at a time. These representations have various favorable properties that allow them to have a very large representational capacity, a good tolerance to noise, and the ability to encode semantic similarity between representations (Hawkins and Ahmad, 2016).

The aim of Lewis et al. (2019) was to keep the capacity, noise robustness, and convergence performance of the previous model while including a way for the neocortex to represent and process allocentric object locations. However, it only incorporated the new functionality into a single column, and not in multiple columns able to vote between each other over neocortical layers 2 and 3, as with the network from Hawkins et al. (2017). A significant benefit of multiple columns is parallel processing of inputs from multiple sensors to enable faster inference. The localization model assumes that the agent knows its own direction of movement relative to the reference frame of the environment. In animals, heading retrieval occurs independent from localization in the brain (Julian et al., 2018), which is needed for determining the direction of movement. The model by Lewis et al. (2019) doesn't include an analog of this operation, and assumes that it receives movement vectors that are inside the object reference frame.

This paper builds on the previous models and addresses two limitations of the network in Lewis et al. (2019) mentioned above. It attempts to show how the learned structure of objects could be used to enable biologically plausible orientation invariant object recognition. To address some of the limitations of previous studies this paper proposes two main changes to current models. First, the output layer equivalent to L2/3 in the neocortex from Hawkins et al. (2017) is implemented to enable the use of multiple columns for the network of Lewis et al. (2019), making it more biologically plausible. Secondly, an orientation layer is implemented to give each column its own representation of object orientation. This layer is implemented within the context of the location representations of Lewis et al. (2019). The presented work aligns with predictions from Hawkins et al. (2019) regarding orientation representations in the neocortex, and proposes a mechanism to represent orientation in each cortical column similar to the function of head direction cells in the hippocampal complex.

## 2. Methods

### 2.1. Model Description

**Figure 1:**
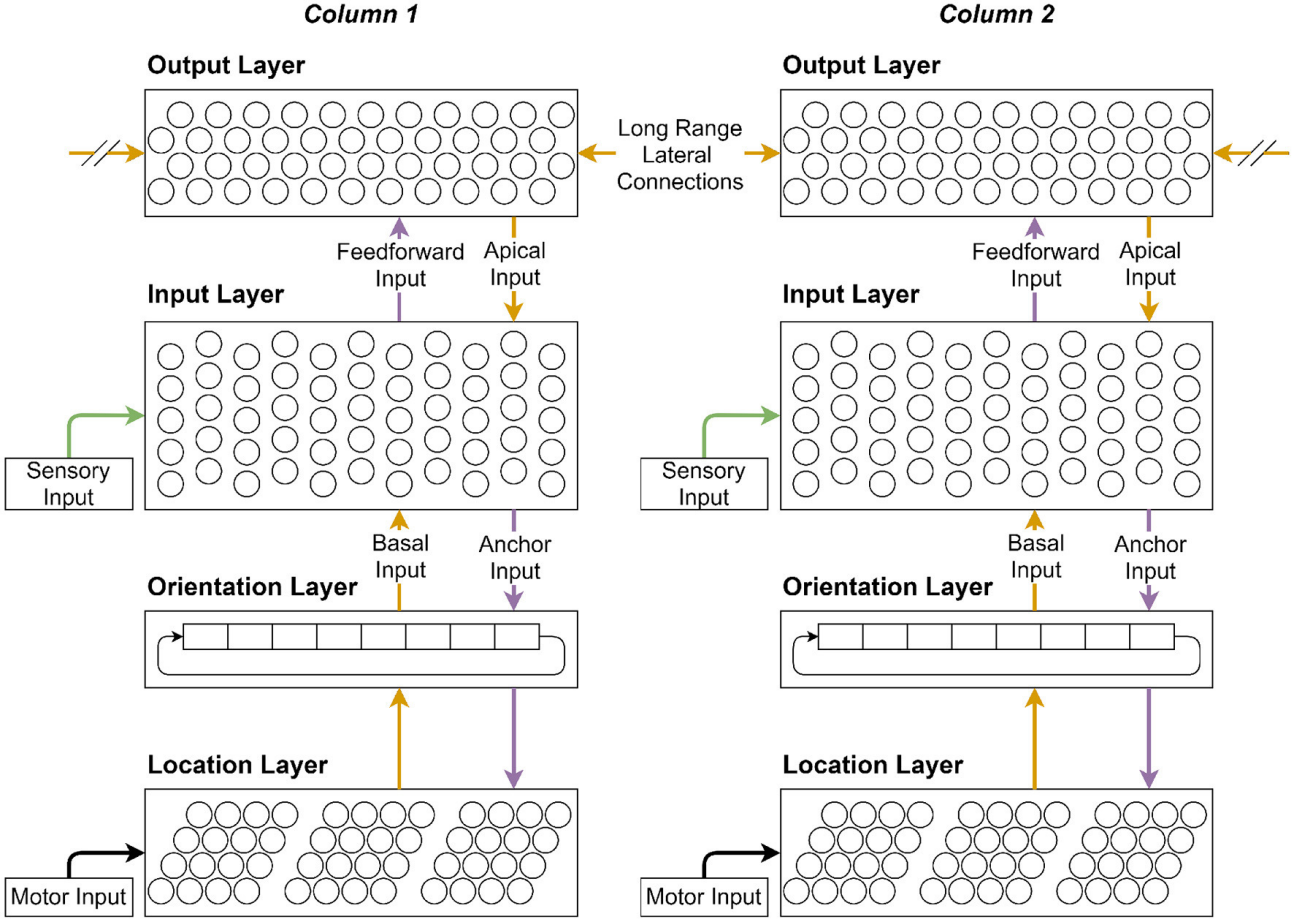
When all the layers are visualized together, they form a stackable cortical column. This figure illustrates the main connections through which various layers interact with each other and themselves. Each cortical column can be connected to an individual sensor that can move independently from other sensors. Each column thus receives its own sensory and motor inputs from its respective sensor. In this case there are two connected cortical columns shown. Connections are color coded to give a visual indication of computation order for the main connections shown in Figure 2.

The proposed sensorimotor network consists of three primary layers and the orientation layer. The orientation layer will be discussed after the primary layers. The network architecture is shown in Figure 1, which can be used as a visual reference for layer descriptions. Starting from the bottom, the first primary layer is the location layer. It uses grid-like representations to translate movements into changes in cell activations, and enable path integration. It also provides movement-based predictions on upcoming sensory inputs as part of the sensorimotor loop. The second primary layer is the input layer. It receives sensory input from the sensory layer, movement-based predictions from the location layer, and top-down expectations from the output layer. It enables predictions to be updated with sensory inputs, and locations to be associated with features. The final layer is the output layer. It learns stable object representations over a stream of feature-location pairs provided by the lower layers. It also consolidates representations from other cortical columns by connecting to other output cells via long range lateral connections in different columns. This enables multiple columns to process different sensory inputs in parallel, and hence infer objects faster and more accurately.

The network activity is calculated over distinct time steps, where each time step denotes multiple sequential processing phases for neural activity. Neurons are either active or inactive at each time step *t*. The location layer consists of grid cell modules, where *bumps* of activity move in relation to sensor movements, assigning a scalar activation value to each neuron at every time step. Neurons in every layer have an activation threshold with multiple independent dendritic segments (Hawkins and Ahmad, 2016). Similar to the internal neuron activation state, dendritic segments have an activation state as well, which can influence whether its respective neuron is active or not. Each segment's activation state is determined by the number of active synapses that is part of its representative set, relative to its activation threshold. With respect to the layer descriptions below, $D_{c,d}^{\text{loc}}$ and $D_{c,d}^{\text{in}}$ are vectors that represent the synapses on dendrite *d* of cell *c* in the location and sensory layers. Synaptic weights are either 0 or 1, and are determined by scalar activation thresholds. Dendrites generally have small numbers of synapses after learning, keeping the vectors sparse to benefit from the advantages of sparse distributed representations (Hawkins and Ahmad, 2016). Through the use of multiple dendritic segments per neuron, robust recognition of independent sparse patterns is possible, which can then be associated with multiple location or sensory environments.

#### 2.1.1. Input and Location Layers

The location layer here is equivalent to the location layer in Lewis et al. (2019), and the input layer is equivalent to the input layer in Hawkins et al. (2017). Within a mini-column *i*, the active cells are defined through the binary array $A_{t}^{\text{in},i}$, where the input layer activity $A_{t}^{\text{in}}$ is defined as the concatenation of all mini-column activities $A_{t}^{\text{in},i}$ in a layer. Similar to the input layer, the location layer activity $A_{t}^{\text{loc}}$ is defined as the concatenation of all module activities $A_{t}^{\text{loc},i}$, for module *i* at time *t*. Contrary to the input layer, a cell in the location layer will be driven to become active if any of its dendritic segments become active. The location layer cell activity is updated after movements (denoted as $A_{t,\text{move}}^{\text{loc}}$), and after sensory-based inputs (denoted as $A_{t,\text{sense}}^{\text{loc}}$). Projections from the location layer to the basal dendritic segments of the input layer is defined through a binary vector $D_{\text{in}}^{c,d}$, which has the same length as $A_{t}^{\text{loc}}$, where 1 represents a connection to a corresponding cell in the location layer. Similarly, projections from the input layer back to the location layer's dendritic segments are defined by vector $D_{\text{loc}}^{c,d}$, which has the same length as $A_{t}^{\text{in}}$, where 1 represents a connection to a corresponding cell in the input layer. Both of these binary vectors are usually very sparse, because they connect to active cells that are already sparse when learning new representations.

Dendritic segments are only activated if they receive sufficient input relative to their activation threshold. The input layer and location layer's state can be calculated using binary vectors $\pi_{t}^{\text{in},c}$ and $\pi_{t}^{\text{loc},c}$, which each show cells with at least one active dendritic segment for that layer. They determine how much each of the layers' cell activity was *predicted* from the cell activity of the other layer. With θ^in^ and θ^loc^ denoting the active dendritic thresholds, $\pi_{t}^{\text{in},c}$ and $\pi_{t}^{\text{loc},c}$ can be calculated as:


(1) $$\begin{array}{l} \pi_{t}^{\text{in},c}=\{\begin{array}{ll} 1, & \exists_{d}\left[D_{c,d}^{\text{in}}\cdot A_{t,\text{ move}}^{\text{loc}}\geq \theta^{\text{in}}\right]\\ 0, & \text{otherwise} \end{array} \end{array}$$

(2) $$\begin{array}{l} \pi_{t}^{\text{loc},c}=\{\begin{array}{ll} 1, & \exists_{d}\left[D_{c,d}^{\text{loc}}\cdot A_{t}^{\text{in}}\geq \theta^{\text{loc}}\right]\\ 0, & \text{otherwise} \end{array} \end{array}$$

If a cell has an active apical segment there are slight modifications to how $\pi_{t}^{\text{in},c}$ is calculated. A slightly lower value for θ^in^ is used in Equation (1). Furthermore, if there are multiple cells predicted in a mini-column, then only the cells with active apical segments (feedback from the output layer) become active. These top-down expectations provided by the current representation in the output layer improves the precision of the current set of cell activations in the input layer.

**Figure 2:**
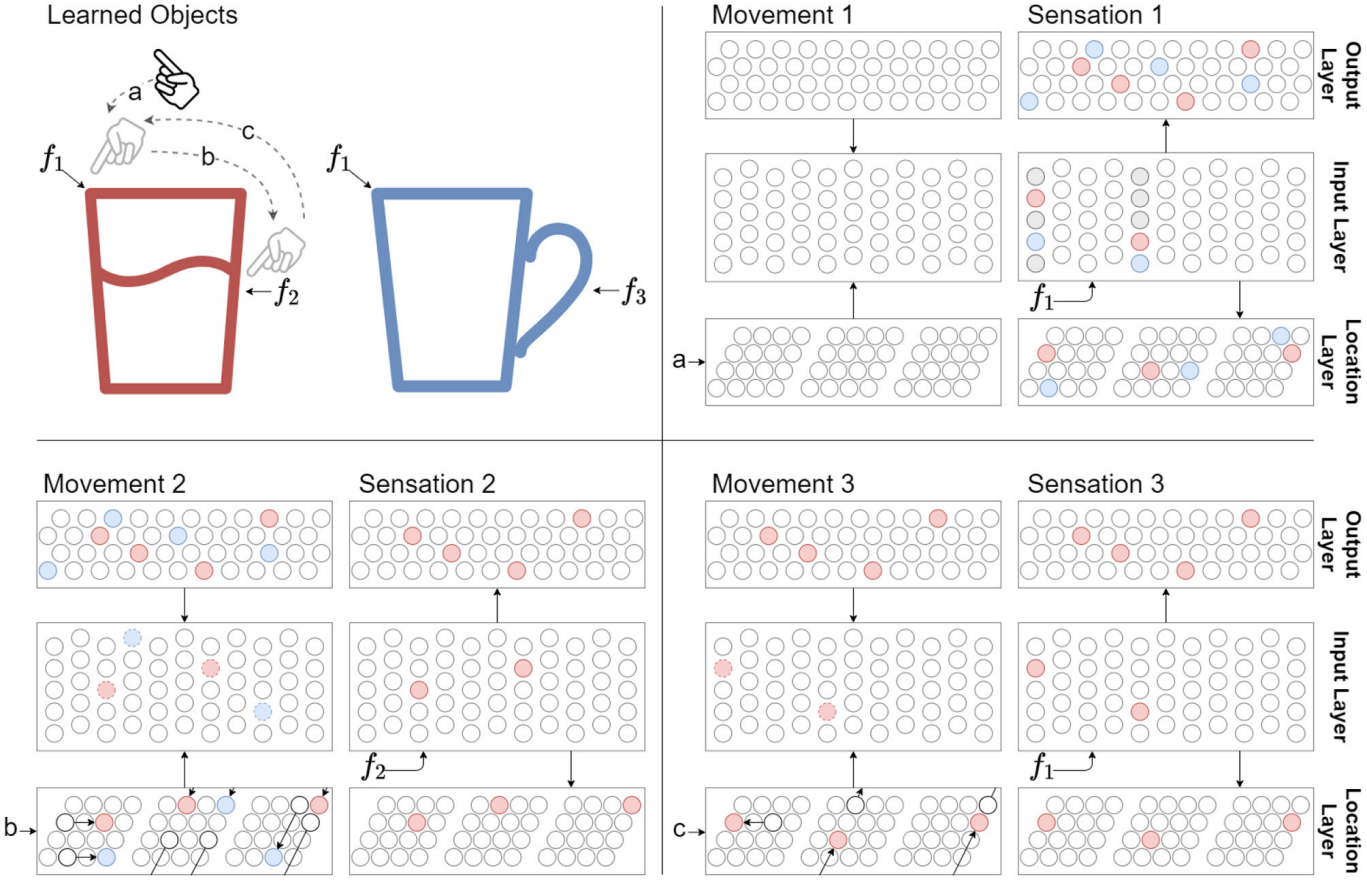
The sensorimotor sequence of a sensor moving across a known object (red glass) is used to recognize the object. Features *f*_1_, *f*_2_, and *f*_3_ represent sensory input when the sensor senses at their respective locations. The distance between these features are traversed by motor commands a, b, and c, which is also used as an input to the network. Cell activity colors are matched to object colors, and connection colors to Figure 1. Three movements and sensations are shown to a network that have already learned the two objects. **Movement 1:** There is no network activity after the first movement command because the network didn't have a previous location representation. **Sensation 1:** The first sensation of feature *f*_1_ causes all cells in the respective mini-columns to activate, since none of the cells were predicted. Note that *f*_1_ has been learned on two objects, thus there are two representations (red and blue) in the input layer for the sensed feature at the current location. These activations drive the two object representations to become active in the output layer, as well as two location representations. **Movement 2:** The location layer performs path integration in response to motor input b on both its location representations. The activity bumps are shifted according to module scale and orientation. The input layer receives modulatory input from the location layer through its basal connections and from the output layer through its apical connections. Note that the top-down expectations are applied on the predictions from path integration. In this simple example these expectations agree with the aforementioned predictions. This modulatory input predicts both *f*_2_ and *f*_3_ for the next sensation. **Sensation 2:** After sensing *f*_2_, only the predicted cells in the respective mini-columns become active. This in turn drives a single object representation in the output layer to become active, as well as a single location representation. The network has now identified the red glass. **Movement 3 and Sensation 3:** Following movements will maintain the unique object representation while the sensed features agree with the predictions from path integration and top-down expectations. In this case movement c back to the start only predicts *f*_1_ in the context of the red glass.

The location layer activations are updated as in Lewis et al. (2019), where a collection of independent grid cell modules, $\Phi_{t,\text{move}}^{i}$, with their own scale and orientation represent the layer. Active cells are always part of a Gaussian bump of cell activations at a certain phase within the module's spatial period. During movement, the bump of activity across modules proportionally change according to each individual module's scale and orientation. The location layer activity, $A_{t,\text{move}}^{\text{loc}}$, is directly updated in response to movements. It is then possible to compute which of the sensory features are predicted by the updated location. The prediction is represented by $\pi_{t}^{\text{in}}$, and denotes the cell activations on distal dendritic segments of the input layer as shown by Equation (1) above. Therefore, $\pi_{t}^{\text{in}}$ has a modulatory effect on input layer activity and is based on the concatenated cell activity in all grid cell modules. It must be noted that due to the *filtering* nature of successive sensations, every plausible sensory feature associated with the current possible locations are predicted for the current time step. This is why it is highly unlikely to recognize an object during the first sensation with a single cortical column for objects that share sensory features, and thus multiple sensations are needed to successfully converge on an object representation as explained in Figure 2.

#### 2.1.2. Input Layer Activity

When the input layer senses features on an object, these sensations are used to evaluate whether the predictions made in response to sensor movements are correct or incorrect. They provide a ground truth, and will activate all possible feature-location pairs when there are no good predictions. This is when unions between representations are both activated and filtered according to modulatory inputs. The sensory layer directly projects to the feed forward inputs (proximal zone) of the input layer, meaning that cells in each mini-column share a receptive field. This enables the same inputs to be associated with different contexts (or cells in a mini-column). The sparse representation that represents the sensory input feature is denoted by $W_{t}^{\text{in}}$. Predicted cells are primed to become active before other unanticipated cells. Correctly predicted cells will inhibit other cells in the mini-column. Only cells with active dendritic segments (predicted) will become active if their associated mini-column was in $W_{t}^{\text{in}}$. If a mini-column in $W_{t}^{\text{in}}$ has no predicted cells, the mini-column bursts and activates all of its cells. This conditional activation for activated cells in the input layer $A_{t}^{\text{in},i,c}$ is defined as:


(3) $$\begin{array}{l} A_{t}^{\text{in},i,c}=\{\begin{array}{l} 1,\;i\in W_{t}^{\text{in}}\text{ and }\pi_{t}^{\text{in},i,c}>0\\ 1,\;i\in W_{t}^{\text{in}}\text{ and }\sum_{c^{\prime }}\pi_{t}^{\text{in},i,c^{\prime }}=0\\ 0,\;\text{otherwise} \end{array} \end{array}$$

After the location layer has exclusively narrowed down the current location out of all the initial possibilities, only one cell in each mini-column in $W_{t}^{\text{in}}$ will be active for the currently sensed feature. Ambiguity is represented in the location layer through a union of activity bumps that are associated with the currently sensed feature. It must be noted that the process of narrowing down possible objects with respect to the current sequence of feature-location pairs involves incorrect sensory input predictions. These predictions from the location layer could potentially not be part of $W_{t}^{\text{in}}$, after which that set of objects will be removed from the pool of possible objects. In other words, this is where the internal model of the world is compared and adjusted to still remain plausible while fitting it to the ground truth sensed from the environment.

#### 2.1.3. Updating the Location Layer

Sensory input is not only used to confirm or deny predictions, but it also feeds back to the location layer as mentioned previously. From Equation (2), $A_{t}^{\text{in}}$ is used to calculate $\pi_{t}^{\text{loc}}$, which actuates activity in the location layer. The list of Gaussian activity bumps in the location layer is updated to match a new set of bumps that were determined by the sensory input. This is the same sensory input that was used to narrow down the location layer's predictions from the previous time step. This phase completes the feedback loop that started from the location layer, to end in updating the location layer's activity from both its own state and new sensory inputs. Each of the cells in the location layer with a connected active dendritic segment causes the module to activate a Gaussian bump centered on that cell. This set of bumps is defined as:


(4) $$\begin{array}{l} \Phi_{t,\text{sense}}^{\mathrm{loc},i}=\{\begin{array}{ll} \left\{{\vec{\phi }}_{c}|c\;:\;\pi_{t}^{\mathrm{loc},i,c}>0\right\}, & \exists_{c}\left[\pi_{t}^{\mathrm{loc},i,c}>0\right]\\ \Phi_{t,\text{move}}^{\mathrm{loc},i}, & \text{otherwise} \end{array} \end{array}$$

Usually the new set of Gaussian activity bumps is closely related to the previous set, because during inference the possible object pool is incrementally narrowed down. The difference is therefore even further attenuated when objects in the possible pool share a significant amount of sensory features. Note that the list of activity bumps is only updated through sensory input during inference, compared to learning where sensory input does not result in an update in the location layer. In this case it simply reduces the phase to $\Phi_{t,\text{sense}}^{\mathrm{loc},i}=\Phi_{t,\text{move}}^{\mathrm{loc},i}$.

#### 2.1.4. Learning

The learning process is characterized by associating locations with sensory features, and associating sensory features with locations, while inference draws on those associations for use in recognition. Equations (5) and (6) shows that associations between layers are encoded in their projections from one to another through their distal dendritic segments. New objects are initialized with random phases for an activity bump in each module. Due to the way that activity bumps are shifted by movements in conjunction with the properties of sparse distributed representations, this initialization provides a unique environment or reference frame that is linked to that specific object. When learning a new object, the inputs consist of chains of movements and sensations, which is used to calculate $\Phi_{t,\text{move}}^{\mathrm{loc}}$ as shown above, while shifting each module's bump proportionally to its orientation and scale with each movement. $W_{t,\text{sense}}^{\text{in}}$ depicts the set of mini-columns that represent the sensory input, which is used by Equation (4) to cause these mini-columns to burst if they don't recognize the inputs (i.e., if it is a new object being learned). This effect will cause all the cells in the respective mini-columns to activate. During bursting, a random cell is chosen from each of the active mini-columns to represent this specific sensory input (within its context) at the current location. In the case where the current part of the object has already been learned, predictions in the sensory layer will be used to select the corresponding cells on existing active segments to learn on, effectively associating previously known inputs to a new context. Each of the active cells in $A_{t,\text{sense}}^{\text{loc}}$ and the selected cells to learn on for the current time step $A_{t,\text{learn}}^{\text{in}}$, forms connections from one of its dendritic segments, *d*′, to each of the active cells in the other layer as shown below:


(5) $$\begin{array}{l} D_{c,d^{\prime }}^{\text{loc}}\;:\;=D_{c,d^{\prime }}^{\text{loc}}|A_{t,\text{learn}}^{\text{in}} \end{array}$$

(6) $$\begin{array}{l} D_{c,d^{\prime }}^{\text{in}}\;:\;=D_{c,d^{\prime }}^{\text{in}}|A_{t,\text{sense}}^{\text{loc}} \end{array}$$

Existing connections on the dendritic segment remain functional when new connections are formed, due to the use of a bitwise OR between the applicable active cells and the selected dendritic segments. Apical segments on selected cells in the input layer are learned using exactly the same rules as basal segments.

#### 2.1.5. Output Layer Activity

This layer is equivalent to the output layer from Hawkins et al. (2017), and its cell activity represents objects. It receives proximal input from the input layer, and modulatory input from other cells in the output layer representing the same object. The modulatory input originates from both other cells in the same cortical column, as well as cells from other columns via long range lateral connections in the same layer. Similar to neurons in other layers, proximal input in the output layer acts as a driver, and modulatory input as a bias. This combination of inputs enable the object representations in the output layer to remain stable over multiple sensations (and movements), and is facilitated by the ability of cells in the output layer to pool over multiple feature-location pairs in the input layer. The output layer also provides feedback to the input layer. The input layer receives this feedback through its apical dendrites, and is able to use it in conjunction with predictions generated from movements to more accurately predict upcoming sensory inputs.

The output layer activity, $A_{t}^{\text{out}}$, is calculated from taking both the feedforward and modulatory distal inputs into account. Similar to the input layer, cells with the best lateral connections from the previous time step, and sufficient feedforward overlap with the input layer, will activate. The feedforward overlap, $o_{t}^{\text{out},c}$, for cell *c* is defined as:


(7) $$\begin{array}{l} o_{t}^{\text{out},k}=\underset{i,c}{\sum }I\left[f_{i,c,k}\geq \theta^{\text{out}}\right]A_{t}^{\text{in},i,c}, \end{array}$$

where *f*_*i,c,k*_ is the synaptic permanence of the synapse between input cell *c* in mini-column *i* and output cell *k*. θ^out^ is the activation threshold, and *I*[] is the indicator function. The indicator function indicates the membership of synapses that are above the activation threshold. The feedforward overlap of output cells can then be used to determine the set of output cells, $W_{t}^{out}$, with enough feedforward input by applying a threshold $\theta_{p}^{out}$ as shown below:


(8) $$\begin{array}{l} W_{t}^{out}=\left\{k|o_{t}^{\text{out},k}\geq \theta_{p}^{out}\right\} \end{array}$$

Output cells are connected via lateral dendritic segments to other output cells. Cells are ranked according to the number of active dendritic segments in the previous time step. A cutoff, $\xi_{t-1}^{out}$, is chosen to select the *s* best cells in this ranking. The number of active dendritic segments in the previous time step, $\rho_{t-1}^{out,c}$, is defined as:


(9) $$\begin{array}{l} \rho_{t-1}^{out,c}=\underset{d}{\sum }I\left[D_{c,d}^{out}\cdot A_{t}^{out}\geq \theta_{b}^{out}\right], \end{array}$$

where $\theta_{b}^{out}$ is the active dendritic threshold. The *s* best candidates can then be determined by applying $\xi_{t-1}^{out}$ to the number of active dendritic segments, and combining it with cells with sufficient proximal input, $W_{t}^{out}$, to determine the activations of output cells, $A_{t}^{out,c}$, as shown below:


(10) $$\begin{array}{l} A_{t}^{out,c}=\{\begin{array}{l} 1\text{ if }i\in W_{t}^{out}\text{ and }\rho_{t-1}^{out,c}\geq \xi_{t-1}^{out}\\ 0\text{ otherwise} \end{array} \end{array}$$

$A_{t}^{out}$ is defined as the concatenation of all output cell activities, $A_{t}^{out,c}$. Note that if there are less than *s* cells that have enough dendritic support in a cortical column, $\xi_{t}^{out}$ would be zero, causing all output cells with sufficient proximal input to become active.

The representations of objects in the output layer are learned by associating a sparse set of cells in the output layer with the collection of feature-location pairs in the input layer that make up an object. Proximal synapses are updated using hebbian-like learning to increase the permanence of active synapses by $p_{ff}^{+}$, and decrease the permanences of inactive synapses by $p_{ff}^{-}$:


(11) $$\begin{array}{l} \Delta f_{i,c,k}=\left[p_{ff}^{+}A_{t}^{\text{in},i,c}-p_{ff}^{-}\left(1-A_{t}^{\text{in},i,c}\right)\right]I\left[f_{ijk}>0\right] \end{array}$$

The basal segments are updated with similar rules as the basal segments from the input layer. The integration of the three primary network layers described above extend the location-based network from Lewis et al. (2019) to support multiple columns, and thus improve its applicability to multi-sensor scenarios while making it more biologically plausible. This provides the architectural context for creating independent orientation representations for each column as described in the following section.

#### 2.1.6. Orientation Invariance

**Figure 3:**
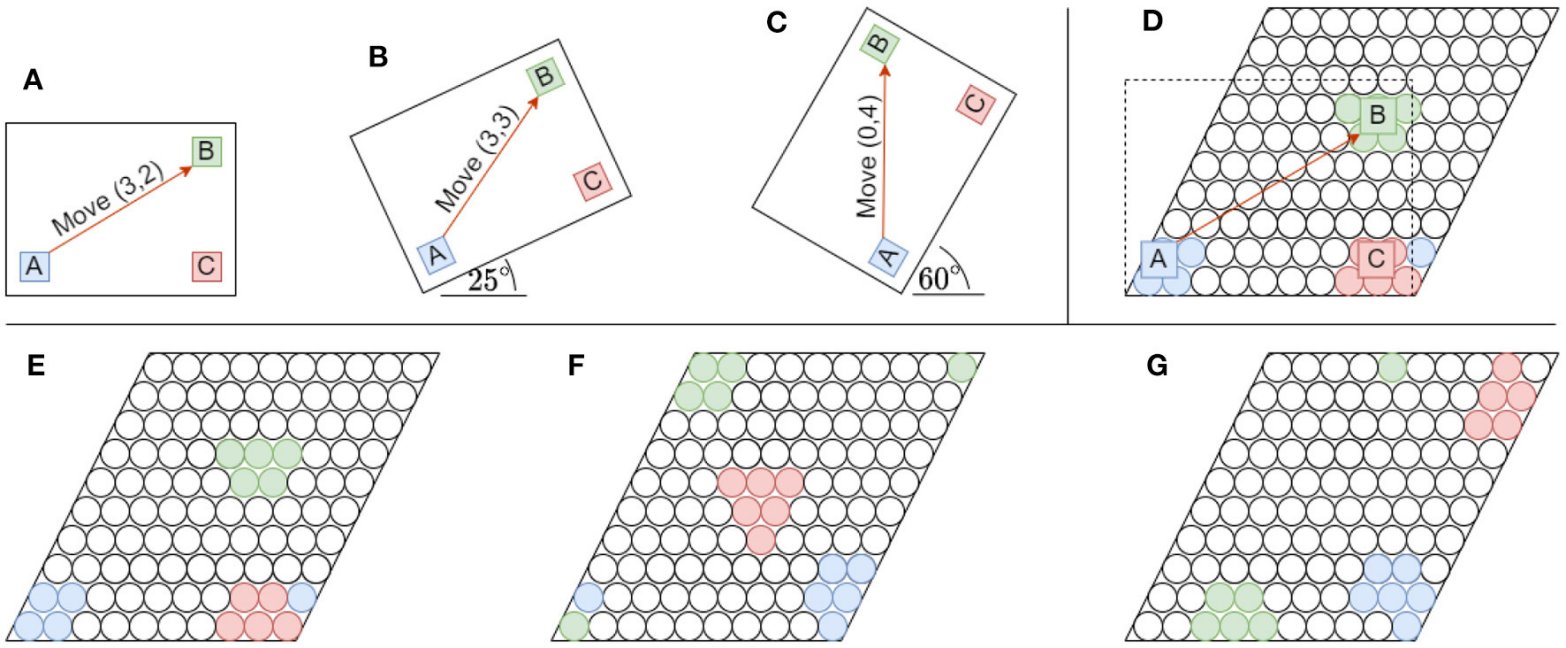
Rotating objects have a significant impact on their underlying representation in the grid cell modules that constitute the location layer. The illustrations show how the representations of features relative to each other change on a single grid cell module. **(A–C)** An object consisting out of three color-coded features (A–C) are shown in three different orientations when rotated around its left-bottom corner. An orange arrow also depicts the displacement (or movement) necessary to move between the same two features (A, B) in all three orientations. The coordinate system used has an origin (*x, y*) = (0, 0) anchored to the lower-left corner of the object. The object scale and movement displacements were chosen for illustrative purposes. **(D)** A [10 × 10] grid cell module showing the receptive fields of the original object anchored to the lower-left corner. The object is slightly scaled up when measuring its grid cell representation for illustrative purposes. Note feature A shown in blue matches receptive fields outside the original grid cell module on the lower left side. Due to tiling, the part outside of the spatial area of the module folds over to the lower right. **(E–G)** The grid cell representations for each of the object rotations in (A–C), respectively are shown from left to right. For illustrative purposes the lower-left corners of both the object and the grid cell module are anchored together. Note how much the relative locations of features distort when they fold over the tiling module while being rotated. These distortions completely disregard the originally-learned relative displacements between object features.

The layers described above only work well for objects that remain in the same orientation during inference as the orientation that they were trained on. The learned shifts of features as a consequence of movements cannot be reused by the network for inference, because the shifts that result from movements between features of rotated objects are very different. Figure 3 demonstrates this limitation, and shows that without a representation for orientation, the network would not be able to use path integration as before.

The addition of the orientation layer solves this problem by considering how the originally learned allocentric representations of objects can be transformed to match the transformed feature-location pairs of rotated objects as a result of rotation. This approach fits in with the concept of mental rotations in humans, and with grid cells that have been shown to represent information about *imagined* movement and spatial orientation, in addition to their traditional role in navigation (Jacobs and Lee, 2016). Grid cell modules can be seen as transfer functions for movements into grid cell representations. Therefore, if you can control the module's orientation you can control how movement shifts internal representations, and hence simulate which change in orientation results in the best fit to original representations.

All grid cell modules will need to be updated in such a way that the resulting representation will be equivalent to the inverse of the object rotation (in order to match with the learned representation), without changing its initial orientation configuration that was fixed during training. This is done by first tuning the modules' orientations before training and inference to form a uniform distribution of all angles in the 360° range. This preset enables modules to be equivalently rotated by simply switching their connections to the next layer in such a way that they will transform the input as if it was rotated. By ordering the modules according to their orientations beforehand, a simple circular buffer can be used to constrain the connections to grid cell modules. This ordering will cause every shift in the buffer to uniformly rotate every module.

This significantly simplifies the identification of the best connection topology for the recognition of a rotated object, while keeping internal configurations of modules intact, enabling them to continue with learning online if needed. The ring buffer is described through the advancement of its indices to new positions. For a buffer size of *N*, the new index, *r*_*i,n*_, that has been shifted by *n* positions is defined as:


(12) $$\begin{array}{l} r_{i,n}=\left(i+n\right)\;\mathrm{mod}\;N \end{array}$$

As an example, the mapping of positions for *N* grid cell modules that have been equivalently rotated by $\frac{360{}^{\circ}}{N}$ degrees (or *n* = 1 positions), can be described as:


(13) $$\begin{array}{l} r_{m}={\left(r_{i,n=1}\right)}_{i=0}^{N-1} \end{array}$$

There are *N* possible orientations for *N* modules, because the circular buffer can be shifted *N* × 1 times before being back in its original configuration. In the example above, the buffer was only shifted *n* = 1 times, and the index started from zero to match algorithmic implementations. Since all possible mental rotations for objects should be considered, a set *R* with *N* evenly spaced mappings for the location layer can be defined as:


(14) $$\begin{array}{l} R=\left\{r_{i,n}|0\leq n\leq N-1\right\} \end{array}$$

*R* therefore contains all connection topologies for grid cell modules in a computationally convenient way. In order to apply and evaluate the modified connections in the context of the network, each module's set of Gaussian activity bumps can be updated according to their new indices. Each module in the location layer keeps a list of activity bumps to reflect ambiguity as mentioned previously. This list gets narrowed down over multiple sensations until only a single location (if inference was successful) is associated with a sensation. There are two main stages in the processing loop where the list of activity bumps is updated. The first is in response to movement ($\Phi_{t,\text{move}}^{\text{loc},i}$), and the second in response to sensations ($\Phi_{t,\text{sense}}^{\text{loc},i}$). When evaluating the plausibility of mental rotations, the cardinality of the set of activity bumps minimizes together with representational ambiguity. Described from another perspective, the firing rate of grid cells in the location layer directly correlate with their convergence on an (possibly rotated) object representation. In the case of movement, the firing rates of grid cell modules in all possible orientations, $\omega_{t,\text{move}}^{r}$, can be defined as:


(15) $$\begin{array}{l} \forall_{r}\in R,\;\omega_{t,\text{move}}^{r}={\left|\Phi_{t,\text{move}}^{\text{loc},r_{i}}\right|}^{-1} \end{array}$$

The same principle can be applied to $\Phi_{t,\text{sense}}^{\text{loc},i}$. The final step is to choose $\omega_{t}^{r}$ with the highest firing rate, and use its respective equivalent rotation as the estimation for the most plausible object orientation to use for classification. Note that the reciprocal is used so that the defined firing rate can be maximized to get the most plausible orientation. Similar to how head direction cells produce orientation-sensitive firing fields, Equation (15) can be used to map the orientational selectivity of the network that converges on an object representation. The most plausible orientation for an arbitrary sensorimotor sequence is chosen after sequentially evaluating each possible object orientation, similar to mental rotations. This could potentially have been implemented in parallel as well to give the same functional result. The sensorimotor sequence that form the current percept of the object is replayed in each orientation to calculate firing rate in the location layer, since movement links feature-location pairs together to form objects.

This fits in with work that has shown different states in processing underlying mental rotations follow each other in identifying objects (Xue et al., 2017). Further work also suggesting that rotation is accomplished in a piecemeal fashion, have shown that people struggle to keep track of the relative positions of multiple features of objects that moved (Xu and Franconeri, 2015). Xu and Franconeri (2015) argue that mental rotations may rely on deeply abstracted representations of objects to make mental rotation computationally feasible within the constraints of neurobiology. One example of this is attentional strategies, where high-spatial ability participants focus on specific parts of the object to increase their mental rotation performance compared to low-spatial ability participants. This ability of using domain-specific heuristics could be linked to the object representation used in the proposed model, where learned sets of feature-location pairs can be both learned and inferred through sensorimotor sequences. This abstraction now just additionally allows for inference of novel object orientations in the proposed model. Using columns could also help explain differences in performance between people in mental rotation tasks, where using more columns in parallel would result in quicker inference for high high-spatial ability participants, and using less columns would result in slower response time (i.e., more sensations required) for low-spatial ability participants as shown by Xue et al. (2017).

### 2.2. Experimental Design

We evaluate the core mechanisms of the model in the context of previous work by Hawkins et al. (2017) and Lewis et al. (2019). To do this, the training and testing environments are designed to evaluate specific characteristics of the model, by creating objects with adjustable complexity and ambiguity with respect to each other and the capacity of the network. This study uses the same method as previous studies (Lewis et al., 2019) for creating the training dataset, and modified it by rotating the learned objects randomly before inference. Objects were generated by modulating the total number of objects, the total number of unique features across all objects, and the number of features per object.

**Figure 4:**
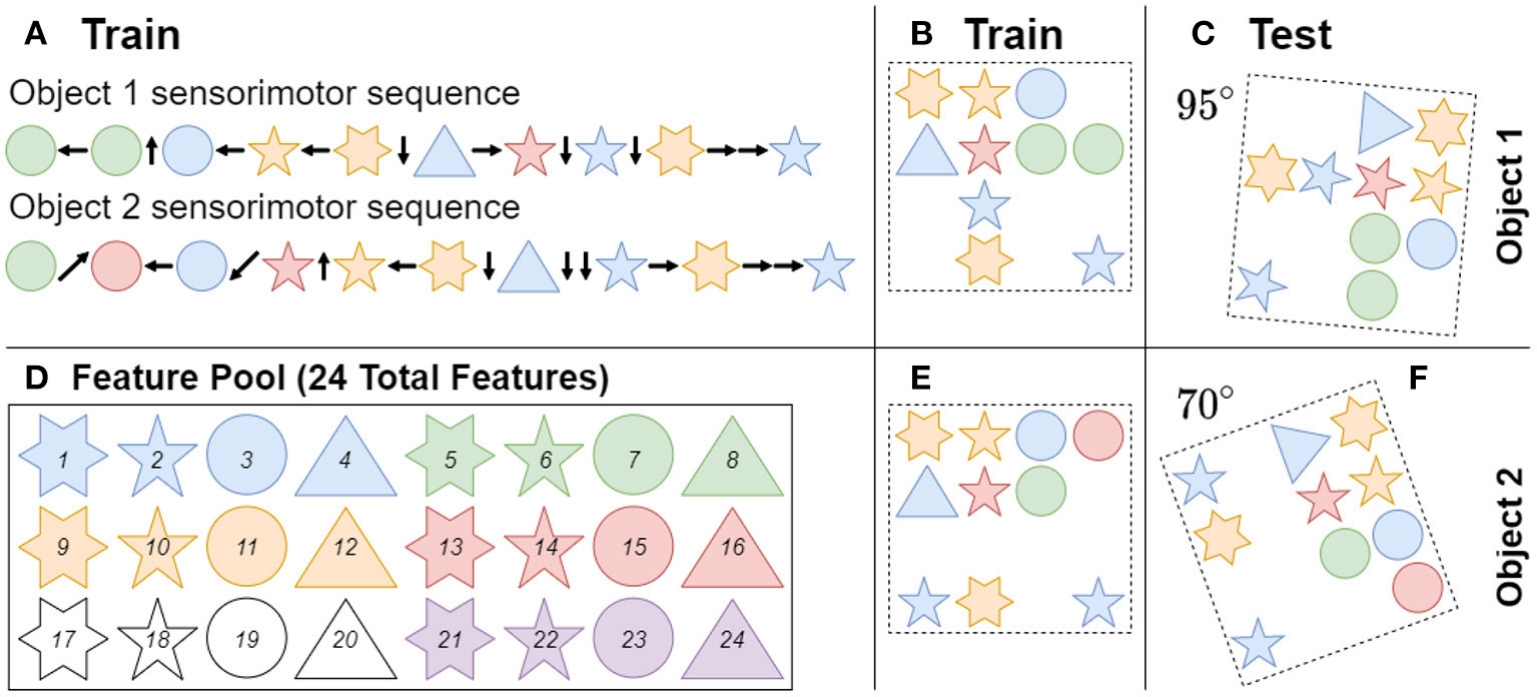
Objects are composed of features sampled randomly from a fixed pool **(D)** with replacement, meaning that objects can share features with each other. Note the shape and color of features only act as a visual aid to show feature uniqueness. Each object is built from 10 features at random locations in a four-by-four grid. Object 1 and object 2 are examples of this process, and were trained in their original orientations **(B,E)**. **(A)** During training the network visits each feature in an object once in a random order, by receiving a sensorimotor sequence. **(C,F)** During inference the network is tested on a randomly ordered sensorimotor sequence from a learned object that has been rotated in a random orientation. When there is no unique feature in an object, the network has to utilize path integration to identify objects through the relative locations of their respective features.

Figure 4 shows an example of how objects are generated and learned through a sensorimotor sequence. Object parameters for the baseline case were chosen to match previous studies to provide a reference performance at 50 total objects with 40 unique features and 10 features per object. For more information on object generation (see Lewis et al., 2019). The current paper accounted for the additional testing dimension of orientation by randomly rotating each learned object a specified number of times to create a novel test class, resulting in a testing set of objects that is significantly larger than the training set. The learned objects were rotated to random orientations using zero order spline interpolation, to keep the features at known values. In cases where multiple iterations for an experiment is done, the random seed for both the network and object sets were varied.

During learning the network visits each feature of an object once, forming a sequence of sensations and movements (sensorimotor sequence) as an input to the network. The goal of the model is to learn an object representation from a single sensorimotor sequence, and then be able to infer the object from a different sensorimotor sequence. Note that the sensed features are converted into sparse distributed representations with the same dimensionality as the input layer. An additional constraint during inference is the maximum allowed number of sensations for recognition (10 by default). In this case, sensations and movements between object features can only occur until this limit is reached. If the object has not been identified within this limit, it is considered a failure. Note that if the maximum number of sensations is more than the number of features on an object, the remaining difference of sensations will revisit object features in a random order with replacement.

Objects are recognized in the output layer when the overlap between the output layer representation and the stored representation for the correct object is above a threshold (set to 30 from Hawkins et al., 2017). The overlaps with stored representations for other objects has to be below this threshold, and the object has to be correctly classified in each cortical column for it to be correctly classified by the network. If the network never converged to an object representation in the output layer, or the converged representation was wrong, or if the output representation was larger than a maximum threshold, the recognition was considered a failure. Note that in the experiments the model always either converged to a single object representation, or did not converge at all.

Other network parameters were also chosen to match models from previous studies (Hawkins et al., 2017; Lewis et al., 2019). The input layer was set to have 150 mini-columns and 16 cells per mini-column. $W_{t,\text{sense}}^{in}$ is comprised out of a predetermined set of mini-columns with a size of 10, representing the object's respective feature at time *t*. The grid cell module orientations in the baseline case were evenly distributed along 60° of possible orientations to match previous studies (Lewis et al., 2019), and the dendritic thresholds, θ^loc^ and θ^in^, were set to 8, respectively. The orientations of grid cell modules were varied across the full range of 360° to evaluate the proposed algorithm. Each grid cell module uses the same scale to match previous work. The output layer was set to contain 4,096 cells, with a threshold for minimum number of active cells set to 40. The activation threshold for proximal dendrites was set to 5, and set to 20 for basal dendrites in the output layer. All parameters were applied to two cortical columns connected through the output layer. These parameters are used in experiments unless otherwise specified in the following sections.

## 3. Simulation Results

**Figure 5:**
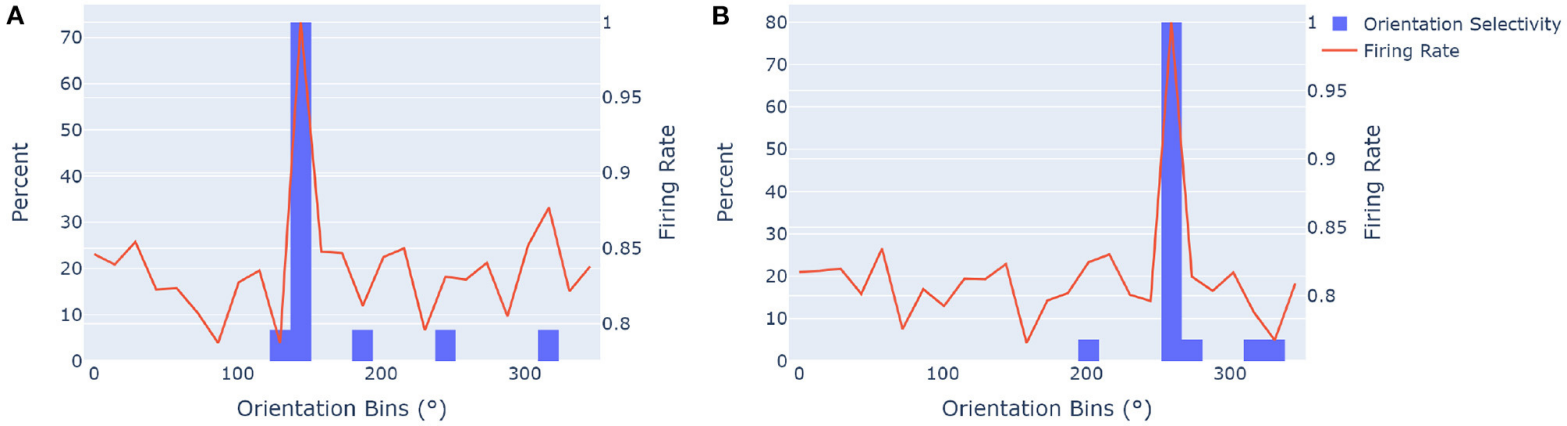
Orientation selectivity is measured by evaluating the percentage of estimated orientations of classified objects that fit into the receptive fields of the ideal model, and how the cumulative firing rate across all objects compare to it. Orientation selectivity is an important part when mentally rotating objects to match their stored representations in order to recognize them. We used a network with 25 grid cell modules and 13 cells per axis. The orientation selectivity was determined for sets of objects rotated by **(A)** 10 and **(B)** 18 positional shifts for the ideal model, and thus associated with the 144° $\left(360\times \frac{10}{25}\right)$ and 259° $\left(350\times \frac{18}{25}\right)$ bins, respectively. The orange trace shows the normalized sum of all firing rates in all the primary directions of rotated modules over the specified sets of objects, giving insight into how the model correctly estimated orientations for most objects. **(A)** The orientational selectivity of an equivalent module rotation of 144° is shown. Approximately 73% of objects that had the highest firing rate in this direction (estimated orientation) fell in the ideal orientational receptive field of this configuration. The normalized firing rate has a primary peak that corresponds with the histogram. **(B)** The orientational selectivity for a module rotation of approximately 259° is shown, with 80% of object orientations correctly identified within the ideal orientation range.

To asses the value of the proposed network, we first evaluate its ability to effectively estimate and represent object orientations. This core computation is based on Equation (15), which was used to evaluate the orientational selectivity of the network in a similar way that head direction cells are evaluated. By summing firing rates in all directions over multiple sensations, the most plausible object orientation over an arbitrary sensorimotor sequence can be calculated. In Figure 5, the receptive fields of an ideal model were used to evaluate the correctness of the receptive fields for the proposed model. This was done with 500 randomly rotated objects from a learned pool of 50 objects with a pool of 40 unique features. From experimentation, 25 grid cell modules and 13 cells per axis gave the network enough capacity and angular resolution to work at a suitable level, and was thus chosen. **Figure 8** further explores the effects of these parameters on model accuracy and capacity. The ideal model has receptive fields that is uniformly centered around each equivalent network rotation. These ideal receptive fields thus form orientation bins that can be used to evaluate the model's orientation selectivity over multiple objects. For example, for 10 grid cell modules there is an angular spacing of $\frac{360{}^{\circ}}{10}=36{}^{\circ}$ between each equivalent rotation step, with a receptive field of $\frac{36{}^{\circ}}{2}=18{}^{\circ}$ above and below each step.

Results show that the proposed algorithm clearly converges to the ideal orientation representation in most cases. The normalized sum of all directional firing rates across objects also collectively agreed with the receptive fields. Note that even when the rotation of objects is not correctly estimated, the network can still infer an object in some occasions, the probability of inference is just lower. The orientational accuracy shown in Figure 5, gives insight into false positive errors for orientation estimation. This is when estimated orientations for objects are wrong with respect to the ideal receptive field that they attempt to embody. In the case of the two illustrations in Figures 5A,B, the percentage of errors are the sum of all estimated rotations outside of the ideal case (26.67% in Figure 5A and 20% in Figure 5B). In the same way, false negative errors can be calculated from the perspective of the ideal orientational receptive fields of rotated modules, by looking at which objects' rotations were different for a given ideal orientational receptive field. Experimentation showed no significant difference between false positive and false negative errors for orientation estimation.

The network has been shown to have comparable directional selectivity to the ideal case, but there was still a significant amount of errors. Head direction firing fields also don't have perfect estimation accuracy (Gaussian-like shape), and experimentation on the proposed model showed a similar trend with most error magnitudes being small, meaning that they lie in the neighboring ideal receptive fields. This raises the question of how these orientation estimations carry over to model accuracy and capacity. The model's spatial representational capacity needs to be high enough to enable the movement projection errors to be within the network's tolerance, or else path integration will destabilize current object representations. Network capacity is thus also important for recognition time, as objects that have lower representational ambiguity can be recognized faster. Accuracy in turn depends on recognition time, as objects that are not identified within a specified number of sensations are considered unrecognized. The base number of features per object is 10, and the base maximum number of sensations is also 10 to match the total number of object features, since each feature should only be sensed once during inference in the default case.

**Figure 6:**
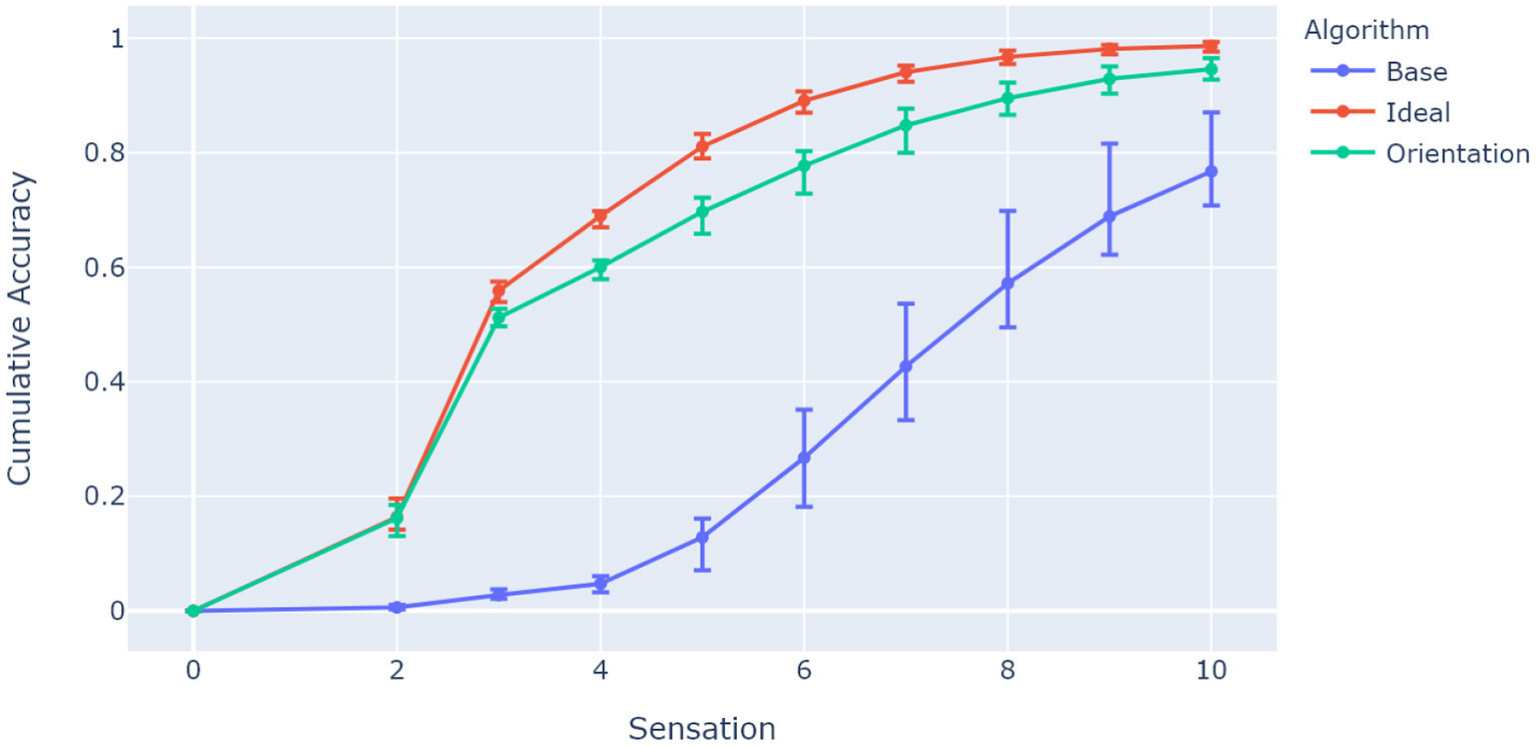
Comparison between algorithms showing model accuracy for 25 grid cell modules and 13 cells per axis. The experiment was repeated 10 times, with the 5th, 50th, and 95th percentiles shown. The base algorithm represents a network with no orientation capabilities, while the ideal algorithm has prior knowledge of the ideal equivalent orientation of its location layer for each object. The accuracy has been tested for 1,000 random rotations from a training pool of 50 objects, and accumulates over 10 sensations as more objects are recognized. The proposed algorithm (*M* = 0.948, *SD* = 0.014) has comparable accuracy to the ideal model for the given parameters, and improves significantly from the baseline case (*M* = 0.780, *SD* = 0.0596), *t*_(9)_ = 11.46, *p* < 0.0001.

Accuracy in this context refers to the fraction of objects classified within a limited number of sensations. Since it is linked to network capacity, it should reflect the differences between the ideal case where orientations of objects are correctly estimated, the base case which assumes the same object orientation in testing and training (which was done in prior models; Hawkins et al., 2017; Lewis et al., 2019), and the proposed network algorithm which uses a mental rotation mechanism to estimate orientation. Figure 6 shows a comparison between these approaches for the same parameters where the previous experiment left off: 25 grid cell modules and 13 cells per axis. The network inferred 1,000 random object orientations from a training pool of 50 objects (20 orientations per object) that share 40 unique features.

**Figure 7:**
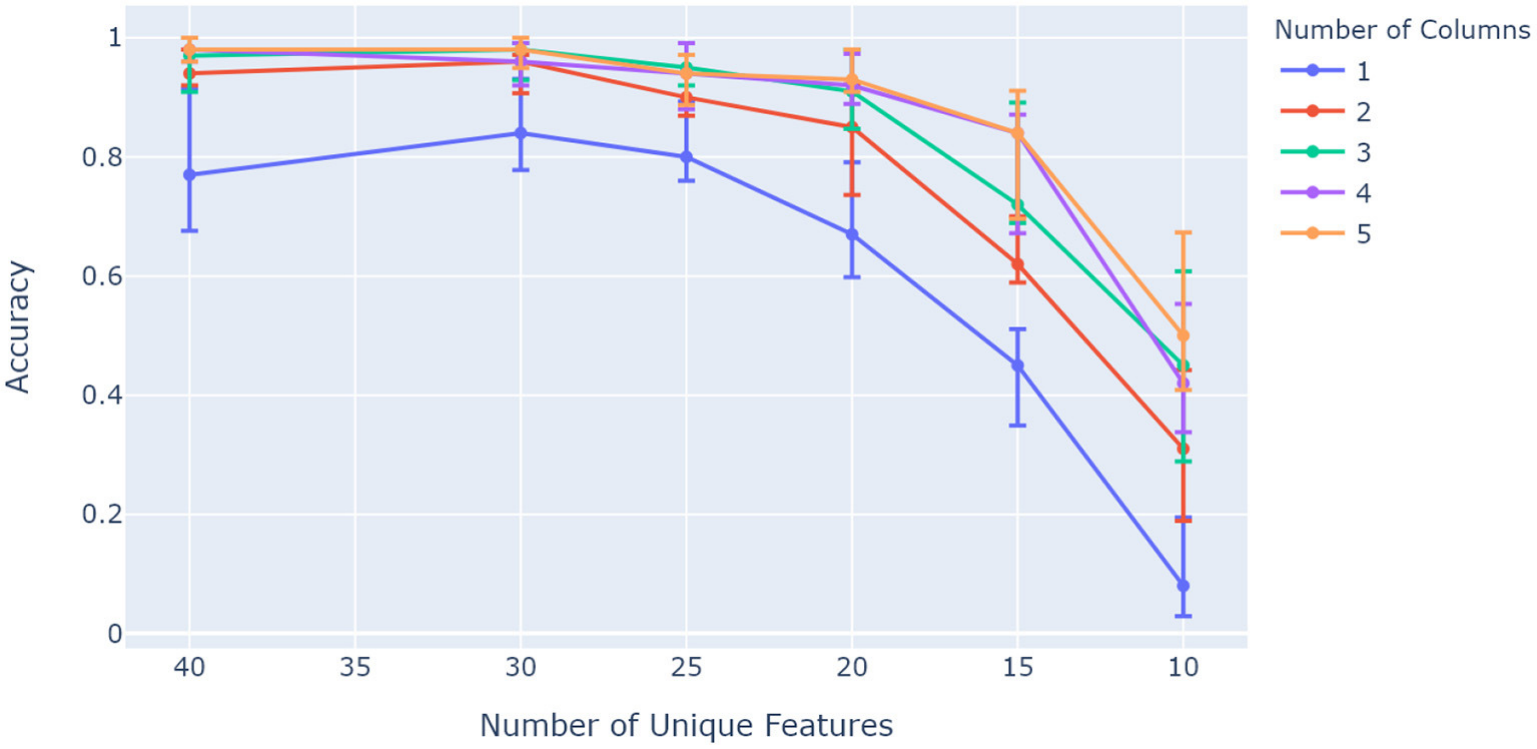
Comparison between different numbers of columns over a range of unique feature pool sizes. More columns retain better accuracy as object ambiguity increases (object uniqueness decreases). The network was configured to have 25 modules and 13 cells per axis, and was trained on 50 objects with 10 features each. The experiment was repeated 10 times, with the 5th, 50th, and 95th percentiles shown.

Figure 6 shows how the ideal case and the proposed orientation algorithm have the capacity to evaluate rotated objects, while the base model struggles to invoke unique representations for the given sensory features and locations. A particular characteristic of interest in the context of contributions for this paper is whether capacity increases with the number of columns as with previous models (Hawkins et al., 2017). This was investigated by evaluating network accuracy while changing object ambiguity for models with different numbers of columns. Object ambiguity was controlled by changing the number of unique features available for a total of 50 trained objects. Figure 7 shows how models with more columns perform better on objects that share more features. This shows that more columns utilize their ability to sense multiple features of objects in parallel, and provide modulatory input to each other via the output layer.

**Figure 8:**
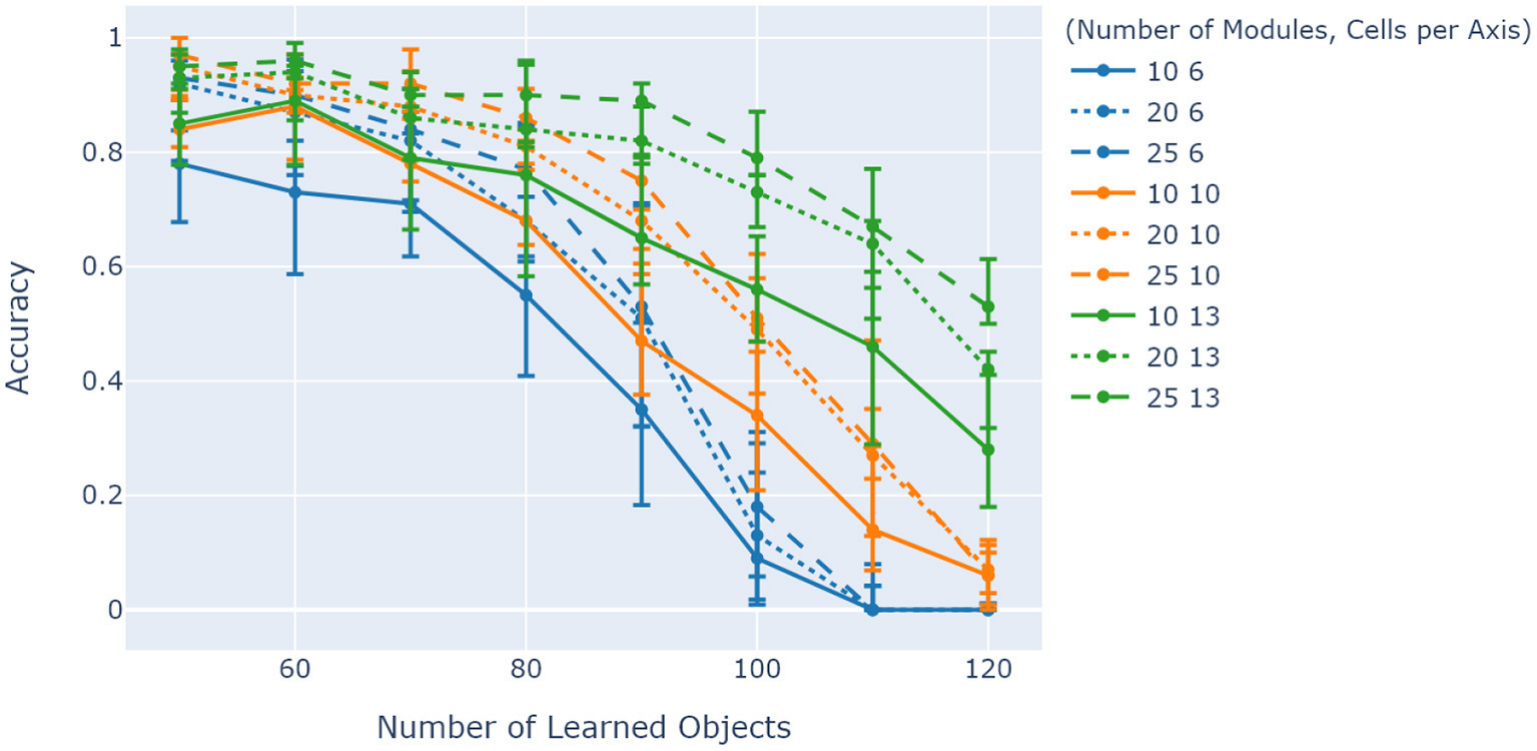
Comparison between the effects of cells per axis and number of modules on model accuracy. Object ambiguity is adjusted by varying the amount of learned objects for a fixed number of unique features of 40. Cells per axis is denoted by color and number of modules is shown by line shape. The illustration shows that generally models with more cells per module have better accuracy for more ambiguous objects, but only if they have enough grid cell modules to counteract the increased projection errors from higher cells per module. The experiment was repeated 10 times, with the 5th, 50th, and 95th percentiles shown.

Evaluation of the model was tied together by considering how its capacity is affected by changing the two main parameters of interest to the orientation layer efficiency: the number of grid cell modules and the cells per module. Figure 8 shows a comparison between the combined effects of these two parameters on network accuracy while increasing object ambiguity through changing the number of learned objects. Initially, the illustration shows a general increase in accuracy from 6 cells per axis to 10 cells per axis, but a more ambiguous difference between 10 and 13 cells per axis. For a given number of cells per module, the number of modules also increases model capacity in general. Comparing the solid lines (10 grid cell modules over various cells per axis), an increase in capacity between 6 and 10 cells per axis is evident, but almost no increase in capacity between 10 and 13 cells per axis (50–70 learned objects) on average.

## 4. Discussion

The proposed network model for sensorimotor recognition extend on models from previous studies (Hawkins et al., 2017; Lewis et al., 2019), by incorporating a novel mechanism for dealing with rotated objects without any additional training. The network consists of three primary layers: a location layer equivalent to the location layer from the proposed model in Lewis et al. (2019), and an input and output layer equivalent to their counterparts from the proposed model in Hawkins et al. (2017). The neural mechanism presented for orientation estimation, and the connection with aforementioned layers creates a model that continues with the vision of previous work to create a biologically plausible framework for artificial intelligence, and forms the main contribution of this paper. Using the same training methods as previous studies (Lewis et al., 2019), the network is able to learn an allocentric object representation by only visiting each feature on an object once. The representation of space through grid cell modules is then utilized to virtually manipulate learned representations to enable inference of learned objects that have been rotated. This is done while keeping the ability to use path integration on these virtual rotations of objects, enabling inference of objects from potentially novel sensorimotor input sequences.

Results from Figure 7 indicated that the model capacity scales with the number of columns, agreeing with previous studies (Hawkins et al., 2017). Generally, increasing cells per module and the number of grid cell modules increased capacity as shown by Figure 8. However, results have shown almost no increase in capacity from 10 to 13 cells per axis at 10 grid cell modules for 50–70 learned objects on average. An explanation for this behavior is that between 6 and 10 cells per axis, the cells per module was the determining factor in accuracy, while between 10 and 13 the number of modules started to become the determining factor. In other words, comparing the solid lines in this illustration shows how these two parameters do not necessarily have separate impacts on model capacity. An increase in number of modules for 10 and 13 cells per axis eventually results in a similar capacity increase expected from previous studies (Lewis et al., 2019).

Results from Figure 8 also show that there is an overlap between traces with different cells per axis for lower degrees of ambiguity. This overlap shows a key transition in object ambiguity compared to model capacity with regards to cells per axis. Note that as the number of learned objects increases, the differences between them decreases. Since there is a fixed number of unique features available, only the relative locations of features on objects can uniquely identify them, meaning that a higher spatial sensitivity will be more beneficial. This is where the number of cells per module start to differentiate models, by enabling them to discern differences in locations much easier. Higher numbers of learned objects start to show this difference (for a given number of modules), with 20 modules for 13 cells per axis even doing better than 25 modules for 10 cells per axis. This highlights an area for parameter optimization for a given set of objects, but that is not the focus of this study.

The model's capacity can increase from various aspects, but results suggest that the location layer is the biggest bottleneck. The number of modules seem to have a significant impact on accuracy, and when combined with enough cells per module, provide a way for the proposed orientation algorithm to reach comparable performance to the ideal model for rotated objects. Note that the model has the same performance as previous studies on unmodified objects (not shown). These results have shown that the model can reliably recognize objects after they have been rotated to novel orientations, while not losing network capacity characteristics compared to previous studies (Lewis et al., 2019).

The orientation mechanism is equivalent to the concept of mental rotations seen in humans when recognizing objects in unseen orientations (Xue et al., 2017). As mentioned in section 2.1.6, mental rotations is when an object is virtually manipulated until it is similar to a stored representation, after which it can more easily be recognized. This mechanism indicates that mental manipulations of objects enable orientations of sensors to be represented relative to the allocentric reference frame of an object. This is comparable to the function of head direction cells, which keeps track of head direction (orientation of the sensor) independent of position. Recently, grid cells have also been shown to be modulated by head direction (Gerlei et al., 2020), suggesting that they could possibly provide downstream processes with local viewpoint information in addition to a representation of space. Furthermore, grid cells have been shown to support imagined movement and spatial orientation (Jacobs and Lee, 2016). Recent theoretical work on intelligence proposes that there exists equivalent cells to grid cells in the neocortex, which support conceptual movement between abstract locations (Hawkins et al., 2019). The proposed model encapsulates these aspects of grid cells involved in mental simulations of object representations (could also be described in terms of top-down controllability). It fits in with a prediction by Lewis et al. (2019) suggesting mechanisms similar to head direction cells for each column in the neocortex, by implementing orientational selective firing fields for equivalent object rotations.

Due to the fixed number of grid cell modules available, the model can only equivalently rotate object representations in a limited number of orientations. It uses its capacity for errors and noise to make up for the orientations between its primary orientations, which is primarily dependent on the number of grid cell modules. This enables the model to use a unique stored representation to infer objects at any possible orientation, by controlling how displacements are transformed to changes in module activity. Conjunctive cells are closely related to grid cells, incorporating multiple navigational signals such as direction and position for path integration mechanisms. Attractor networks are popular for modeling path integration with these cells, and have been shown to predict a periodic distribution of conjunctive cell preferred directions (Keinath, 2016). Keinath (2016) found that cells were tuned in periodic increments, with a lower bound of 10° and an upper bound of 120° between preferred directions. These results resonate with the aforementioned primary orientations of the proposed model, indicating that conjunctive cells could be a biological correlate for equivalently rotating an orientation reference (which produces such periodic distributions of preferred directions). A more detailed direct investigation of biological correlates and mechanisms suggested by the model is a topic for future research.

While the model does enable sensor orientations relative to an object reference frame without any modifications to its internal representations or training method, it does not come without any trade-offs (no free lunch). There are considerably more computations when evaluating the plausibility of possible orientations of an object. Compared to previous studies (Hawkins et al., 2017; Lewis et al., 2019) the computational complexity scales an additional *O*(*n*) times for *n* grid cell modules, since there are *n* equivalent rotations to be considered compared to a single orientation assumed by previous models. Another consideration to consider, is that rotated objects aren't necessarily guaranteed to perfectly match the orientational receptive fields of modules. This means that there will always be a minor movement projection error during path integration (on average). The network will need enough capacity to deal with the magnitude of these errors, which can mainly be mitigated by adding more grid cell modules. This is shown through the comparison between algorithms in Figure 6, where even the ideal case didn't reach perfect accuracy. In this case the proposed algorithm followed closely below it. The biological plausibility of this model is in the same context as previous work with aspects such as the number of grid cell modules used. Parameters thus were chosen to evaluate the model through testing its capacity. As mentioned in section 2.1.6, the proposed circular buffer mechanism was chosen to be simple and computationally efficient. Module orientations don't necessarily have to be equally spaced in all directions, and mappings can be learned in future work aiming to follow biology more closely.

The proposed network extended existing mechanisms to support orientation. This mechanism has been shown to work reliably on novel object orientations, and provide a theoretical implementation of predictions of previous studies within the context of previous work (Hawkins et al., 2019; Lewis et al., 2019). It proposes orientation selective cells for object representations in each column of the neocortex (as it is defined in this and previous models). These cells provide a reference between sensor orientation and learned allocentric object representations.

Future work for this model could lead in various directions. Fixed rotation intervals could be improved by learning orientations of objects using memory mechanisms similar to those used in other layers, or by adding attentional mechanisms to focus on more specific orientation intervals. Scale invariance is still an open question in these allocentric object representations, and would be an ideal addition to orientational invariance. Object compositionality is another natural direction to take in order to increase the capacity for more complex objects and environments, which reflect the hierarchy of the natural world. The model was described using 2D grid cell modules to learn 2D objects, but the neocortex is able to learn 3D objects. The proposed model should work for 3D if its grid cell representations support 3D allocentric locations. Extending grid cell representations to support 3D is also a possible area of future research. There is also scope on the application of the model to real world problems, which would involve implementing algorithms from a high performance computing perspectiveor adding a spatial pooler (Cui et al., 2017) to convert real world sensor data into sparse distributed representations to account for changing input statistics from sensor drift for example.

## Data Availability Statement

The raw data supporting the conclusions of this article will be made available by the authors, without undue reservation.

## Author Contributions

KR conceived the presented idea, developed the theory, performed the simulations, and wrote the manuscript. In addition to general support throughout the study, DH provided insight into neuroscience literature, and helped to write the manuscript. All authors contributed to the article and approved the submitted version.

## Funding

This work was based on the research supported in part by the National Research Foundation of South Africa (Grant Number: 46712).

## Author Disclaimer

Opinions expressed and conclusions arrived at, are those of the author and are not necessarily to be attributed to the NRF.

## Conflict of Interest

The authors declare that the research was conducted in the absence of any commercial or financial relationships that could be construed as a potential conflict of interest.

## Publisher's Note

All claims expressed in this article are solely those of the authors and do not necessarily represent those of their affiliated organizations, or those of the publisher, the editors and the reviewers. Any product that may be evaluated in this article, or claim that may be made by its manufacturer, is not guaranteed or endorsed by the publisher.
